# Supplementary material for: Compensatory Transcriptional Response of Fischerella thermalis to Thermal Damage of the Photosynthetic Electron Transfer Chain
Source: Molecules. 2022 Dec 3;27(23):8515. doi: 10.3390/molecules27238515 (PMC9740203; doi:10.3390/molecules27238515)
Supplement: Supplementary file 1 [file molecules-27-08515-s001.zip › supplemental_figures.pdf]

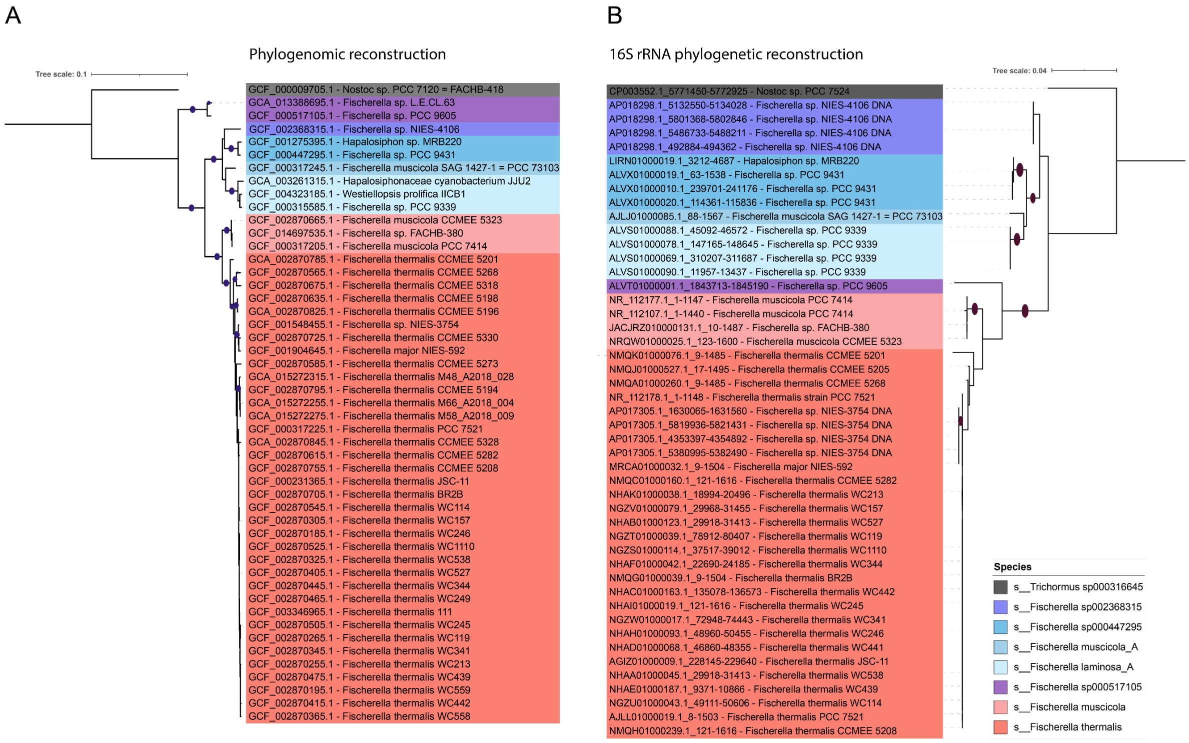

Figure S1. Phylogenetic reconstruction of the genus *Fischerella* by 120 concatenated universal bacterial markers and the 16S rRNA subunit. (A) Phylogenomic reconstruction of 48 Fischerella genomes and one outgroup (*Nostoc* sp. PCC 7120). Bootstrap support values over 90% are shown as black dots in tree nodes. (B) 16S rRNA phylogenetic reconstruction of 49 *Fischerella* 16S rRNA gene sequences and one outgroup. Not all genomes available in the NCBI database have their respective 16S rRNA subunits present in the assemblies. Tree nodes are colored at species level according to (A). Bootstrap support values over 95% are shown as black dots in tree nodes

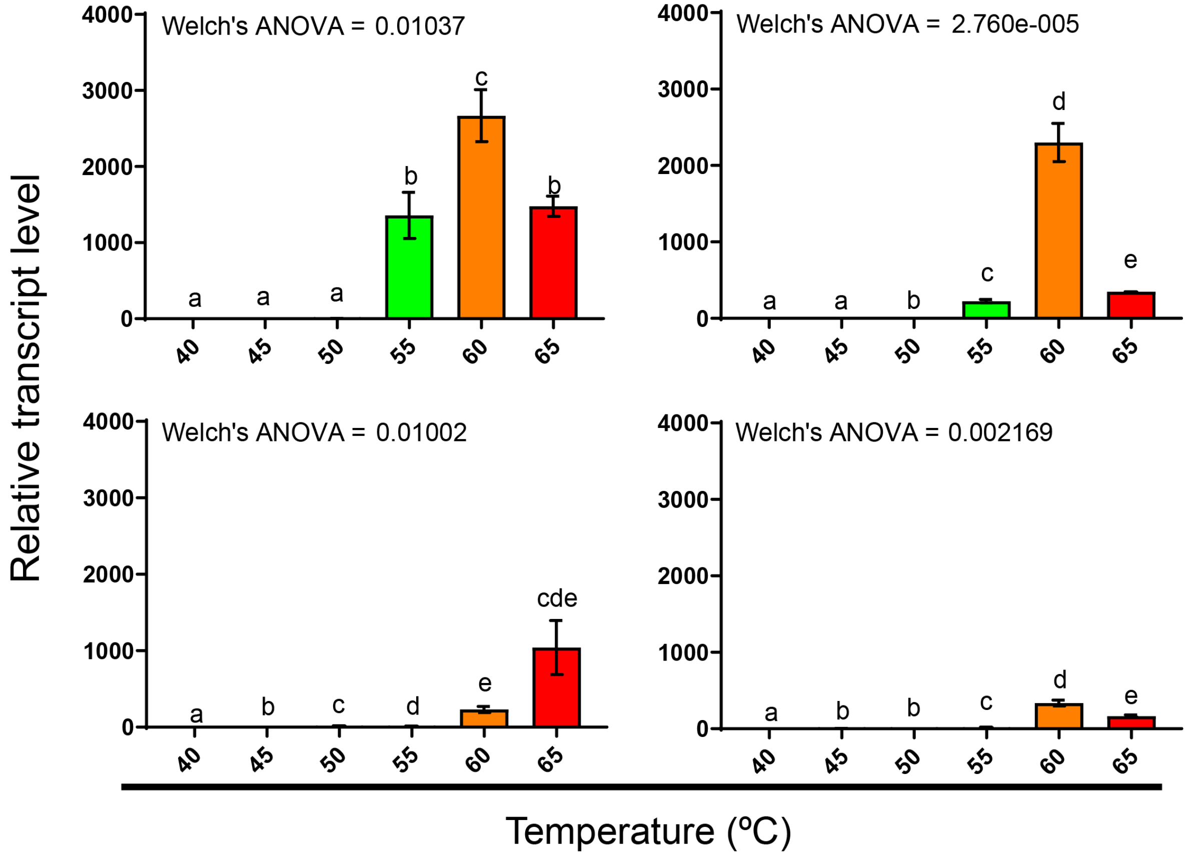

Figure S2. Transcriptional activity of *gro*EL in *F. thermalis* at different temperatures. The transcriptional levels of *gro*EL in *F. thermalis* cultures treated at temperatures from 40-65°C (n=3) were analyzed by RT-qPCR. Relative transcript levels were adjusted to 1 using 40°C as a reference for each analyzed time. Error bars denote the Standard Error of the Mean. Barplots are colored for aesthetic purposes. Welch's ANOVA was used for statistical analysis.

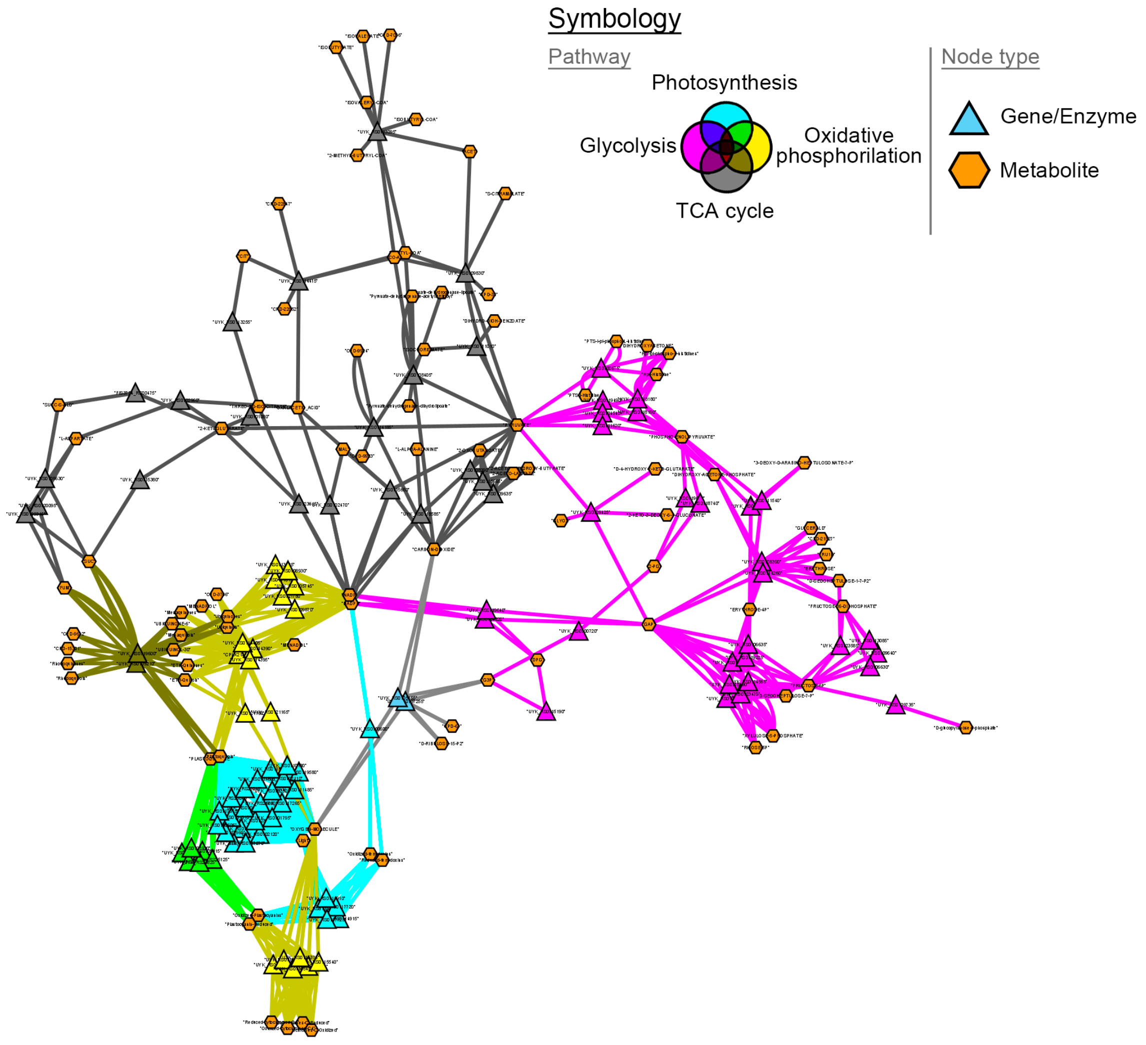

Figure S3. Energy metabolism of *F. thermalis*. Based on gene annotation of *F. thermalis* and their respective enzymatic reactions available in the BioCyc database, the metabolic network of *F. thermalis* was reconstructed. Link color denotes the corresponding metabolic pathway, with intermedial colors indicating association to more than one pathway.
