# Supplementary figures and images for: Compensatory Transcriptional Response of Fischerella thermalis to Thermal Damage of the Photosynthetic Electron Transfer Chain
Source: Molecules. 2022 Dec 3;27(23):8515. doi: 10.3390/molecules27238515 (PMC9740203; doi:10.3390/molecules27238515)

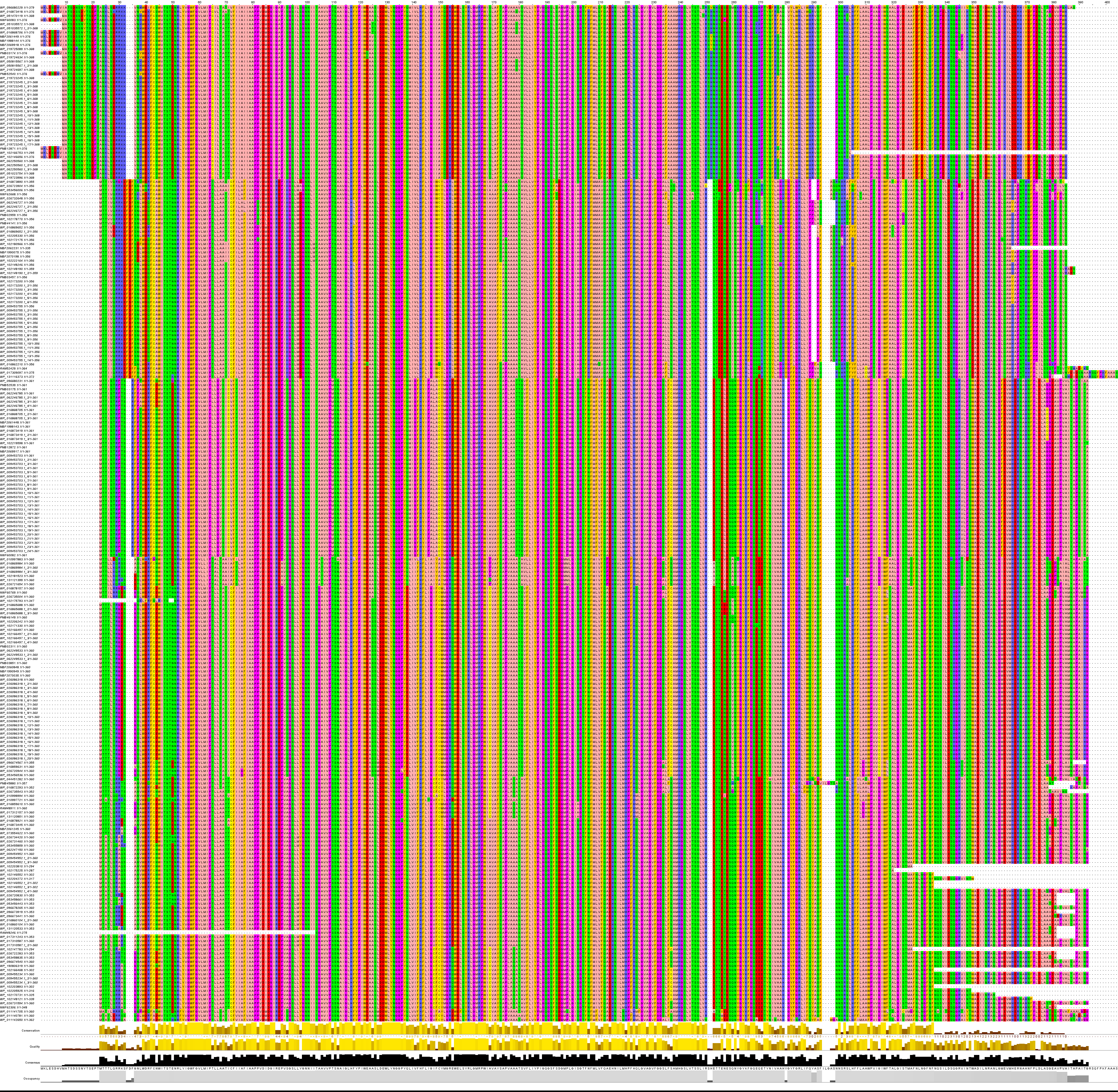

Supplement: Supplementary file 1 [file molecules-27-08515-s001.zip › alignments/jalview/alll_psba_RED.png]

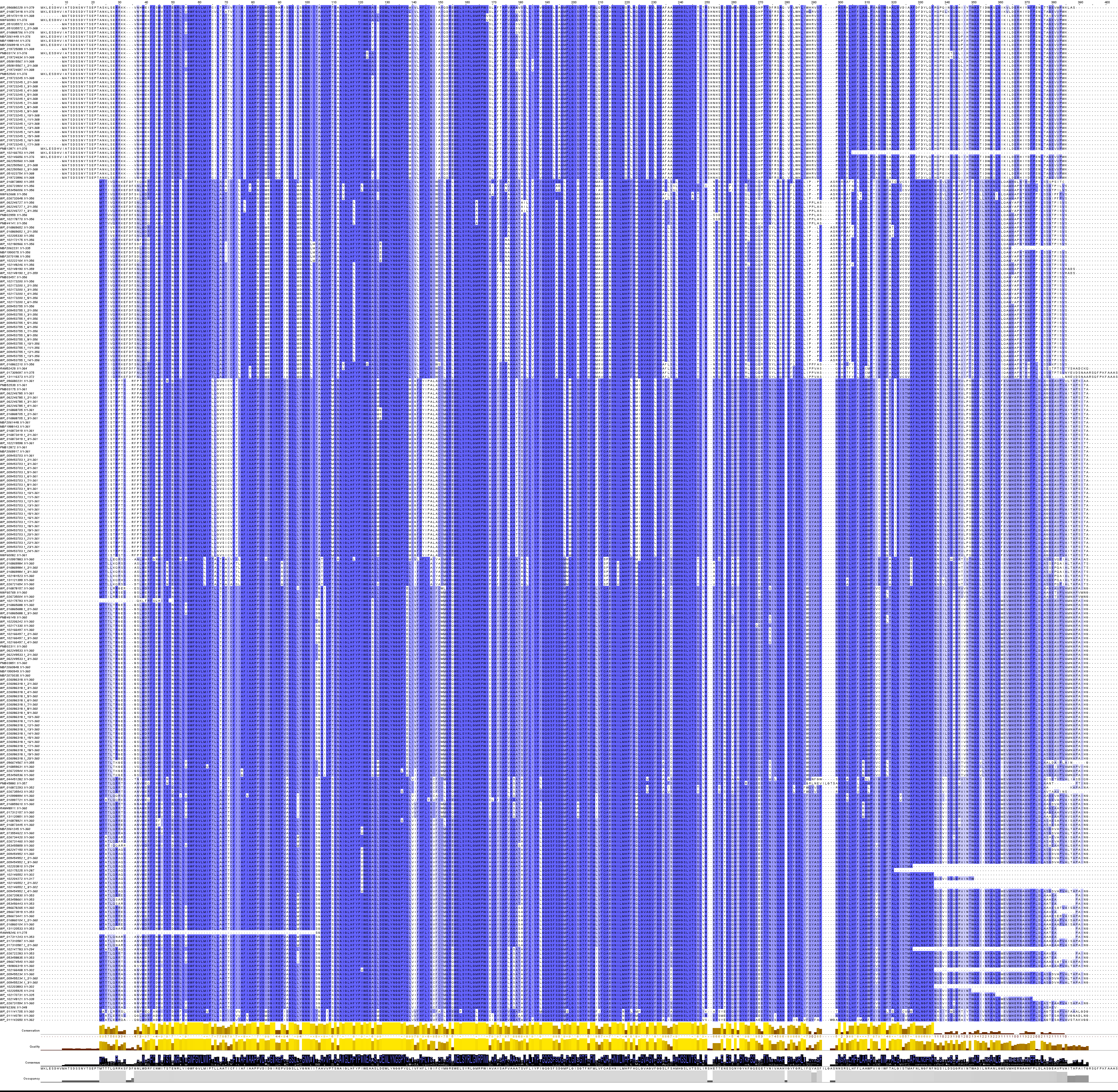

Supplement: Supplementary file 1 [file molecules-27-08515-s001.zip › alignments/jalview/alll_psba_RED_conserv.png]

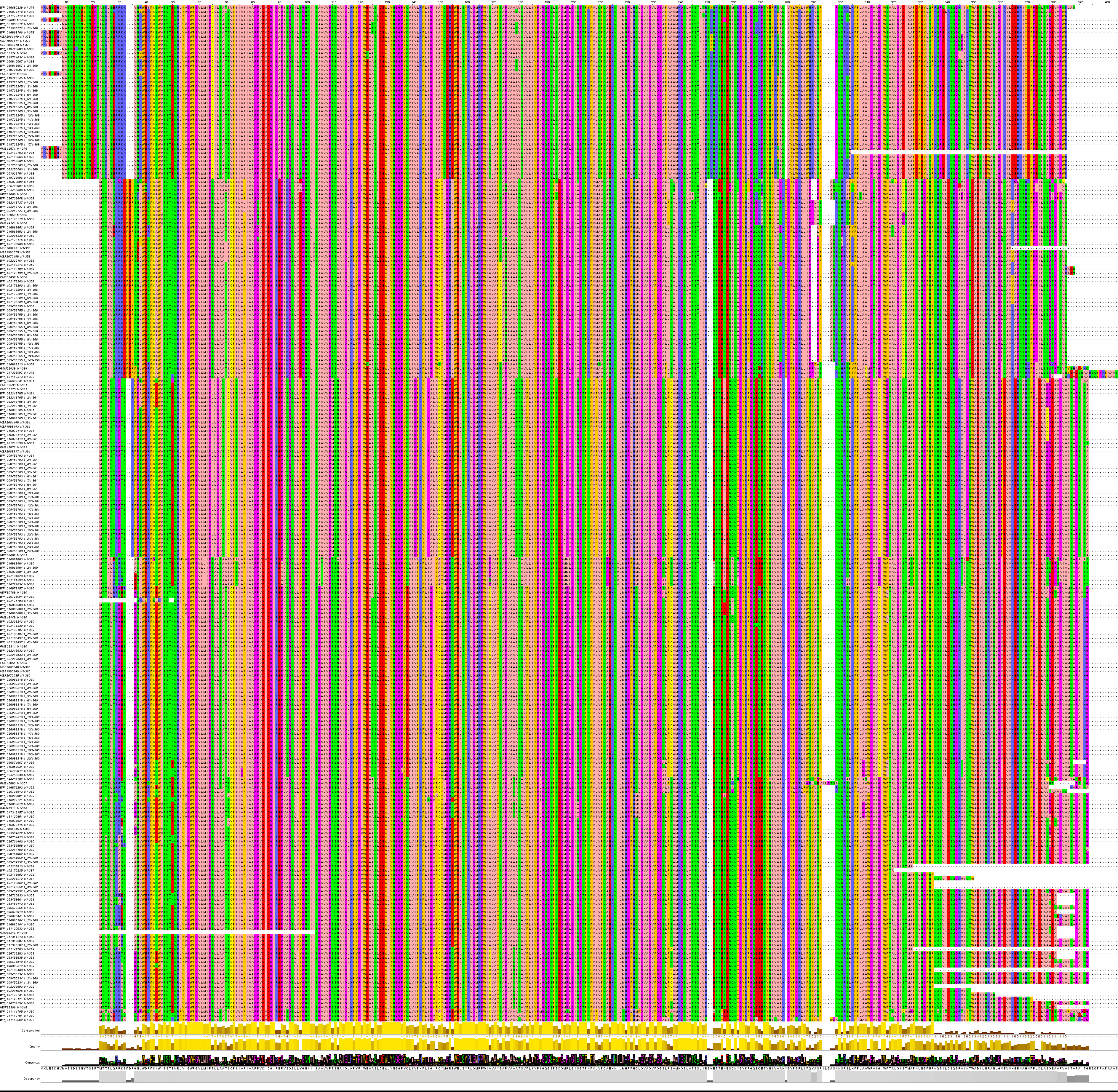

Supplement: Supplementary file 1 [file molecules-27-08515-s001.zip › alignments/jalview/alll_psba_RED_LOGO.png]

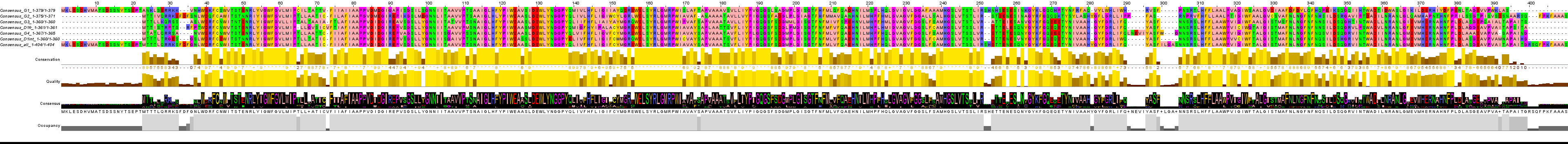

Supplement: Supplementary file 1 [file molecules-27-08515-s001.zip › alignments/jalview/consensus_align.png]

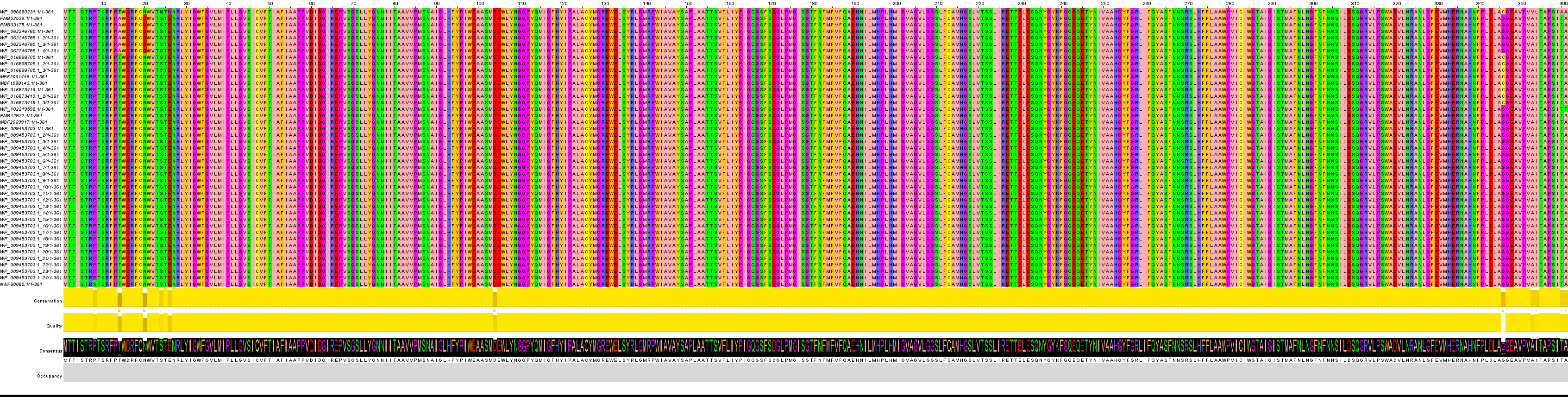

Supplement: Supplementary file 1 [file molecules-27-08515-s001.zip › alignments/jalview/D1fr_RED.png]

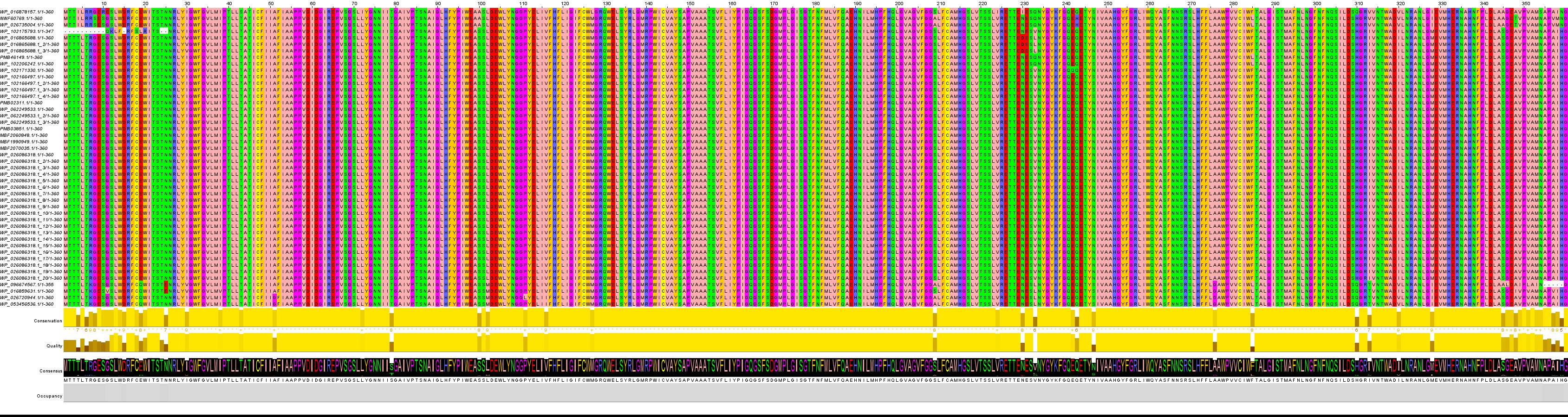

Supplement: Supplementary file 1 [file molecules-27-08515-s001.zip › alignments/jalview/D1int_RED.png]

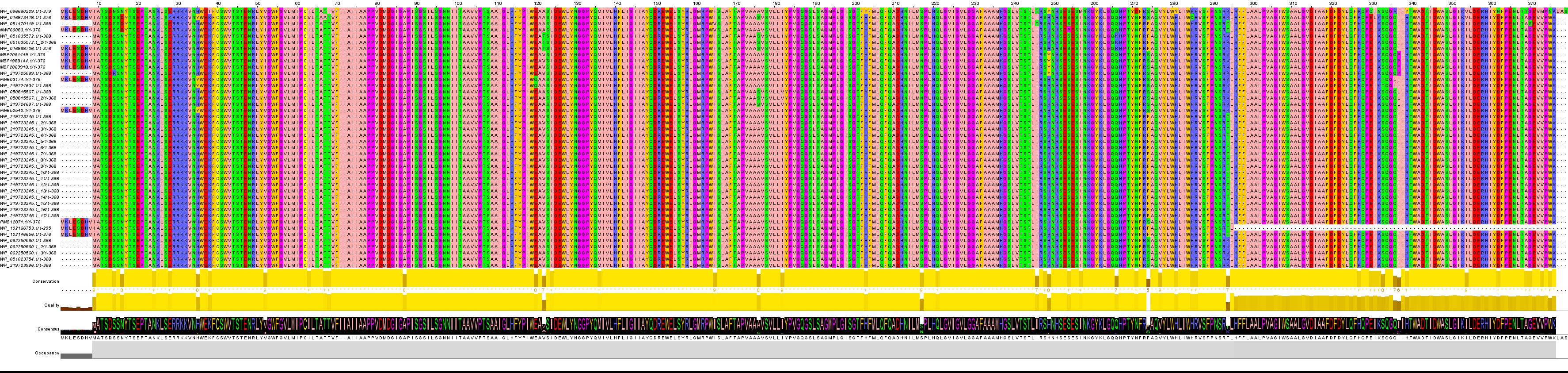

Supplement: Supplementary file 1 [file molecules-27-08515-s001.zip › alignments/jalview/G1_RED.png]

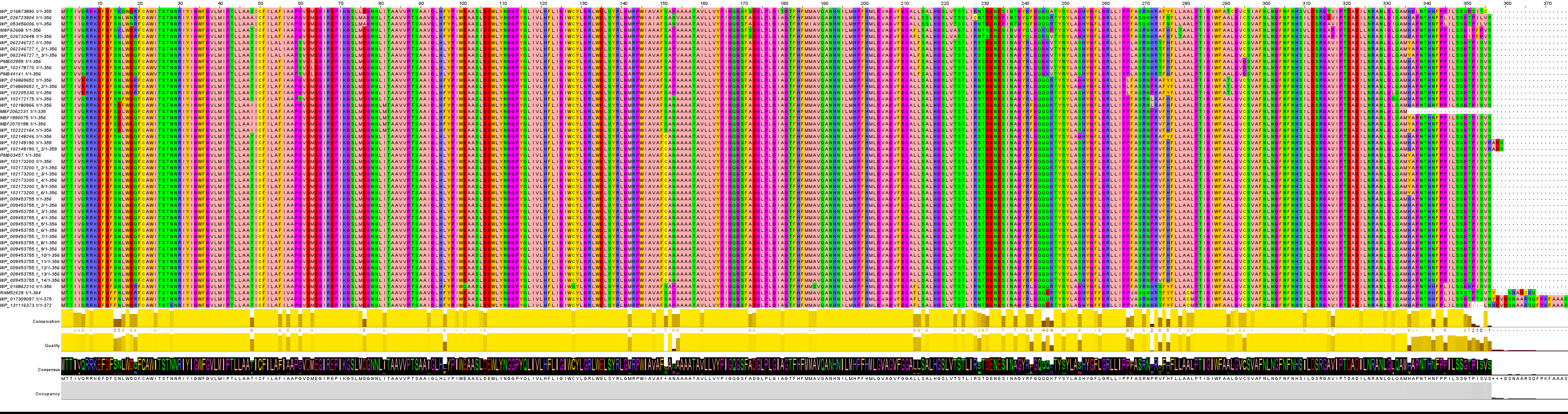

Supplement: Supplementary file 1 [file molecules-27-08515-s001.zip › alignments/jalview/G2_RED.png]

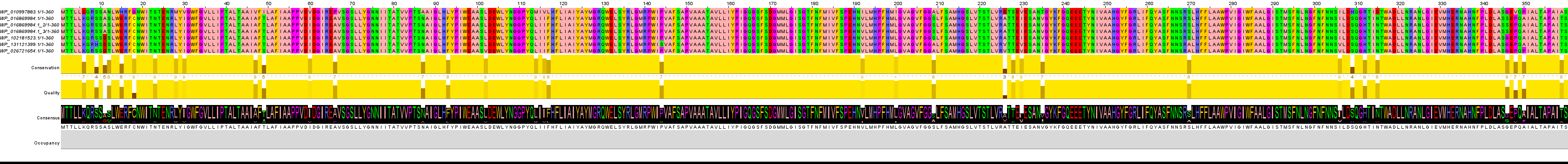

Supplement: Supplementary file 1 [file molecules-27-08515-s001.zip › alignments/jalview/G3_RED.png]

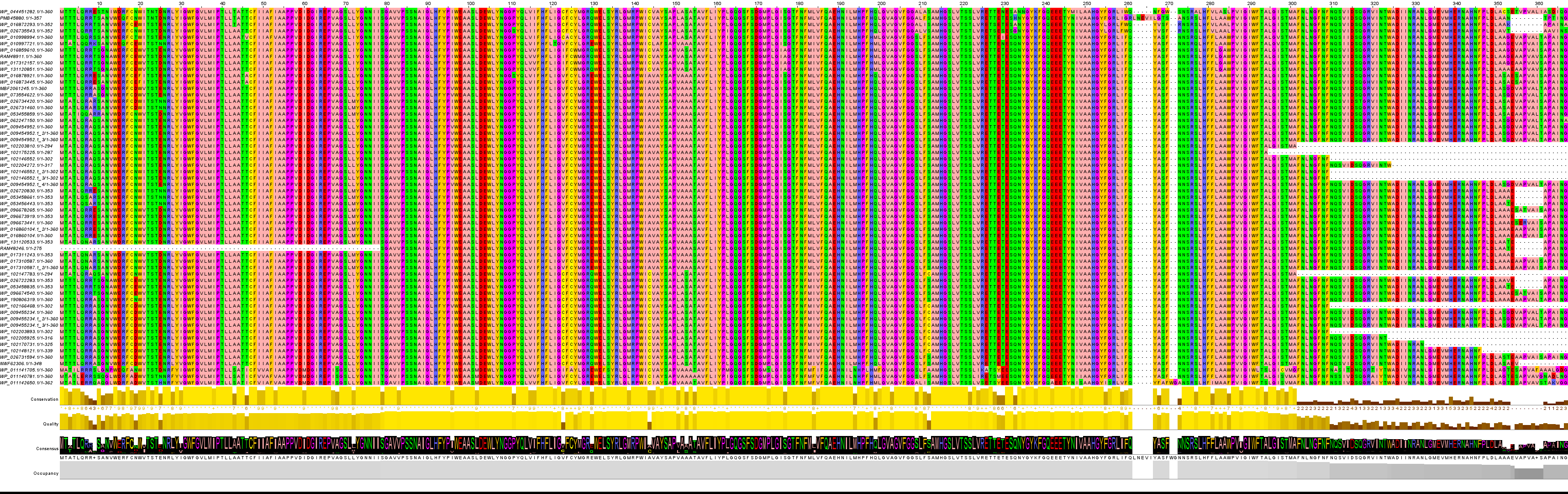

Supplement: Supplementary file 1 [file molecules-27-08515-s001.zip › alignments/jalview/G4_RED.png]
